# Deleterious effects of dialysis emergency start, insights from the French REIN registry

**DOI:** 10.1186/s12882-018-1036-9

**Published:** 2018-09-17

**Authors:** Michel Alain, Pladys Adelaide, Bayat Sahar, Couchoud Cécile, Hannedouche Thierry, Vigneau Cécile

**Affiliations:** 1 CHU Pontchaillou, Service de néphrologie, 2 rue H Le Guilloux, 35033 Rennes cedex, France; 2 EHESP, Département d’Epidémiologie et de Biostatistiques, Rennes, France; 3 Université Rennes 1, UMR CNRS 6290, Rennes, France; 4 EA MOS EHESP, Rennes, France; 5 Registre REIN, Agence de la biomédecine, La Plaine Saint Denis, France; 6 Faculté de médecine de Strasbourg, Hôpitaux universitaires de Strasbourg, 1 place de l’Hôpital, 67091 Strasbourg cedex, France; 7 Université de Rennes 1, 2 av prof L Bernard, 35000 Rennes, France; 8 Inserm (Institut national de la santé et de la recherche médicale), IRSET, U1085, SFR Biosit, 9 Avenue du Professeur Léon Bernard, 35000 Rennes, France

**Keywords:** Emergency start, Survival, Outcome, ESRD, Dialysis, France

## Abstract

**Background:**

Emergency start (ES) of dialysis has been associated with worse outcome, but remains poorly documented. This study aims to compare the profile and outcome of a large cohort of patients starting dialysis as an emergency or as a planned step in France.

**Methods:**

Data on all patients aged 18 years or older who started dialysis in mainland France in 2012 or in 2006 were collected from the Renal Epidemiology and Information Network and compared, depending on the dialysis initiation condition: ES or Planned Start (PS). ES was defined as a first dialysis within 24 h after a nephrology visit due to a life-threatening event. Three-year survival were compared, and a multivariate model was performed after multiple imputation of missing data, to determine the parameters independently associated with three-year survival.

**Results:**

In 2012, 30.3% of all included patients (*n* = 8839) had ES. Comorbidities were more frequent in the ES than PS group (≥ 2 cardiovascular diseases: 39.2% vs 28.8%, *p* &lt; 0.001). ES was independently associated with worse three-year survival (57% vs. 68.2%, *p* = 0.029, HR 1.10, 95% CI 1.01–1.19) in multivariate analysis. Among ES group, a large part had a consistent previous follow-up: 36.4% of them had ≥3 nephrology consultations in the previous year. This subgroup of patients had a particularly high comorbidity burden. ES rate was stable between 2006 and 2012, but some proactive regions succeeded in reducing markedly the ES rate.

**Conclusion:**

ES remains frequent and is independently associated with worse three-year survival, demonstrating that ES deleterious impact is never overcome. This study shows that a large part of patients with ES had a previous follow-up, but high comorbidity burden that could favor acute decompensation with life-threatening conditions before uremic symptoms appearance. This suggests the need of closer end-stage renal disease follow-up or early dialysis initiation in these high-risk patients.

**Electronic supplementary material:**

The online version of this article (10.1186/s12882-018-1036-9) contains supplementary material, which is available to authorized users.

## Background

Differently from recent trends in the USA [1] and in overall European Union [2], the number of incident patients with End-Stage Renal Disease (ESRD) in France continues to progress, with a steadily rise of 2.2% per year between 2006 and 2012, mainly due to diabetes 2-related ESRD [3].

Preparing patients for renal replacement therapy (RRT) is a challenge for nephrologists whose role is to convince them about the asymptomatic end stage of a vital organ, help them choosing the most appropriate RRT modality, prepare a dialysis access, manage anemia and nutritional support, and assess their suitability for renal transplantation waitlisting. Late referral to nephrologists (defined as a referral &lt; 3–4 months before RRT initiation) has been associated with poorer outcome, prolonged initial hospitalization, higher risk of all-cause death [4–7], and increased costs for the health care system [8]. In France, strong efforts have been made to promote the early referral of patients with chronic kidney disease (CKD), including information to general practitioners about CKD, definition of national guidelines for renal care and referral, and implementation of health networks between hospitals and general practitioners [9].

Once referred, current guidelines recommend delaying dialysis initiation until the occurrence of uremic symptoms, degradation of nutritional status, uncontrolled hypertension, volume overload, threatening acid-base or electrolytes disorders [10]. For some patients, acute pulmonary oedema or threatening electrolytes disorders appear before uremic symptoms, that lead to start dialysis in emergency conditions. The impact of starting dialysis in emergency conditions (“Emergency Start” (ES)) has not been widely studied. A previous French epidemiologic study [11] based on the 2006 Renal Epidemiology and Information Network (REIN) data, pointed out that ES was associated with a worse one-year survival rate than “Planned Start” (PS) of dialysis (74.2% for ES vs. 87.4% for PS, *p* &lt; 0.001). Some other studies also have reported worse outcomes associated with unplanned dialysis start [12, 13], but no strong data is available about the profile of patients starting dialysis in emergency conditions, and the factors leading to ES. Moreover, the group of patients exhibiting an ES is probably heterogeneous and deserve to be better described, in order to enhance their management.

The aim of this study was to compare the clinical status and outcomes of dialysis incident patients in 2012 mainland France according to the dialysis initiation condition (ES or PS), and to analyze the ES group. In addition, patients who started dialysis in 2006 or 2012 were compared to determine whether the ES rates, profile and outcomes changed during this interval.

## Methods

### Study population

This study was based on data from the REIN registry. This registry started in 2002, and progressively extended to the 22 metropolitan French regions and 5 overseas territories. All French patients with ESRD are registered, including those who undergo preemptive kidney transplantation. Patients with acute kidney injury requiring dialysis are not included in the registry. If unclear, chronic kidney failure is defined by a persistent requirement of dialysis after 45 days of RRT (REIN guidelines).

All incident patients aged 18 years or older who started long-term RRT in one of the 22 metropolitan regions in 2012 (or in the 16 regions included in 2006) were included, if the dialysis initiation status (PS or ES) was described. Patients who underwent preemptive kidney transplantation and patients on dialysis after loss of a functional transplant were not included because they were not considered as incident patients (REIN guidelines). Patients from overseas territories were excluded because of the significant differences in demographic characteristics (higher rate of diabetes and hypertension) and clinical practices compared with mainland France.

### Collected data

The proportion of ES among the included patients was calculated, globally and for each region. ES was defined as a first dialysis session within 24 h after a nephrology visit, for life threatening conditions, including acute pulmonary edema, severe hyperkalemia or acidosis, uremic confusion or pericarditis. This applied also to patients with a previous follow-up and presenting with an acute complication. However, the exact cause of dialysis start was not recorded in the registry.

For both groups (ES or PS), the following baseline (i.e., at dialysis initiation) data were collected: age, sex, primary renal disease, nutritional status, comorbidities, walking disability, and ESRD management (place of care, modalities). The number of previous nephrology visits within 1 year before dialysis start was also registered. Primary renal diseases were grouped in three categories, depending on the form of renal function impairment: acute nephropathy (including CKD exacerbation or flare), slowly progressive nephropathy, or unknown (see Additional file 1).

Then, the same data were compared between ES patients who started dialysis in 2006 and in 2012 (in the same 16 regions included in the REIN registry in 2006). Only, the number of nephrology visits in the last year before dialysis was not compared because this item was not recorded in the registry in 2006.

### Statistical analysis

Patients’ baseline characteristics were expressed as frequencies and percentages for categorical variables, and as median and interquartile values (IQR) for continuous variables. Demographic and clinical features were described by subgroups and compared using the Chi-square test, according to the initiation timing and the year of dialysis start: ES vs PS in 2012; ES in 2012 vs ES in 2006. Moreover, subgroups of 2012 ES patients were also distinguished and compared depending on the number of nephrology visits within 1 year before dialysis start (if available): no previous visit vs ≥3 visits. Missing data were presented in tables for the descriptive results when &gt; 10%. Before the analyze of each event of interest, missing data were handled by multiple imputation method.

#### Three-year survival and cox regression

All patients who started dialysis in 2012 were included in the analyses (three-year follow-up was completed for all included patients). Patient survival was assessed from dialysis initiation up to 3 years after dialysis initiation. Kaplan Meier survival curves were plotted for each group and log-rank tests were used to compare three-year survival of the groups. The Cox regression method was used to evaluate the association between patients’ characteristics and three-year survival. All variables associated with the outcome in the unadjusted model (*p* &lt; 0.2) (were included in the adjusted model. All variables with a *p*-value &lt; 0.05 in the final adjusted model were considered as statistically significant. To deal with the problem of missing data, the Multiple Imputation by Chained Equations (MICE) procedure [14] was used for each variable before Cox regression. The process was repeated for all variables with missing values and to stabilize the results, the procedure was repeated for ten cycles to produce a single imputed dataset. Finally, the whole procedure was iterated five times to obtain five imputed datasets.

Statistical analyses were performed with the Stata 13.1 software (College Station, Texas, USA).

## Results

### Comparison of the 2012 incident patients depending on the dialysis start condition (ES or PS)

A total of 8839 patients were included and represented 91% of all incident patients in mainland France in 2012 (9% were excluded because the dialysis initiation status was missing). Among them, 30.3% experienced an ES. The baseline characteristics of the ES and PS groups are presented in Table 1. There was no significant difference in sex and age between the ES and PS groups. Patients experimenting an ES had significantly more comorbidities: 39.2% had ≥2 cardiovascular diseases compared with 28.8% in the PS group (*p* &lt; 0.001), and there was a higher proportion of smokers and respiratory disease, cirrhosis, or cancer in ES group. BMI repartition showed a higher proportion of extreme rates, and a larger part of ES patients had a serum albumin concentration &lt; 30 g/l. The first RRT technique for patients with ES was almost always hemodialysis (98.2% for ES vs 86.6% for PS, *p* &lt; 0.001) and required most often a central venous catheter placement at initiation (85.4% vs 43.3%, *p* &lt; 0.001).

**Table 1:** Characteristics of dialysis incident patients in mainland France in 2012, according to the dialysis initiation condition: planned start or emergency start

	Planned start (*n* = 6161; 69.7%)	Emergency start (*n* = 2678; 30.3%)	
	*n* (%)	*n* (%)	p
Men	3918 (63.6)	1736 (64.8)	0.268
Age, y (mean ± SD)	67.9 ± 18.8	67.8 ± 16.4	0.65
Primary renal disease			&lt; 0.001
Acute nephropathy	478 (7.8)	407 (15.2)	
Slowly progressive nephropathy	4454 (72.3)	1668 (62.3)	
Unknown	1229 (19.9)	603 (22.5)	
Serum albumin &lt; 30 g/dl	981 (15.9)	669 (25)	&lt; 0.001
Missing	1090 (17.7)	563 (21)	
BMI (kg/m^2^)			&lt; 0.001
&lt; 18.5	254 (4.1)	158 (5.9)	
18.5–25	1908 (31)	865 (32.3)	
&gt; 25	2658 (43.1)	987 (36.9)	
Missing	1341 (21.8)	668 (24.9)	
Smoking status			0.006
Current smoker	594 (9.6)	277 (10.3)	
Former smoker	1464 (23.8)	658 (24.6)	
Never smoker	3209 (52.1)	1237 (46.2)	
Missing	894 (14.5)	506 (18.9)	
Respiratory disease	725 (11.8)	457 (17.1)	&lt; 0.001
Hepatic disease	127 (2.1)	80 (3)	0.005
Active malignancy^a^	636 (10.3)	394 (14.7)	&lt; 0.001
Diabetes	2459 (39.9)	1105 (41.3)	0.229
Type 1	162 (6.6)	47 (4.3)	0.006
Type 2	2280 (92.7)	1051 (95.1)	
Cardiovascular diseases^b^			&lt; 0.001
0	2995 (48.6)	1055 (39.4)	
1	1390 (22.6)	573 (21.4)	
2	895 (14.5)	479 (17.9)	
≥ 3	881 (14.3)	571 (21.3)	
Mobility			&lt; 0.001
Totally dependent for transfers	216 (3.5)	209 (7.8)	
Need assistance for transfers	638 (10.4)	407 (15.2)	
Walk without help	4718 (76.6)	1760 (65.7)	
First dialysis modality			&lt; 0.001
HD/HDF	5333 (86.6)	2628 (98.2)	
APD/CAPD	828 (13.4)	49 (1.8)	
Vascular access at initiation			
Central venous catheter	2115 (34.3)	2286 (85.4)	&lt; 0.001
Arteriovenous fistula (used or not)	3291 (53.4)	566 (21.1)	&lt; 0.001
Pre-dialysis anemia management			
Hemoglobin &lt; 10 g/dl	2660 (43.2)	1628 (60.8)	&lt; 0.001
ESA therapy	2860 (46.4)	709 (26.5)	&lt; 0.001
Residual eGFR ml/min/1.73m^2^ (median, IQR)	9.2 (7.2–12.1)	8.05 (5.5–11.4)	&lt; 0.001
Missing	725 (11.8)	355 (13.3)	
Waitlisted at dialysis initiation (&lt; 80 years)	580 (12.1)	37 (1.8)	&lt; 0.001

^a^ Active malignancy: solid tumors or hematological malignancies

^b^Cardiovascular diseases: myocardial infarction, arrhythmias, coronary insufficiency, heart failure, arteritis of the lower limbs, cerebrovascular accident

*BMI* Body Mass Index, *HD* hemodialysis, *HDF* hemodiafiltration, *APD* automated peritoneal dialysis, *CAPD* continuous ambulatory peritoneal dialysis, *ESA* erythropoietin stimulating agent, *eGFR* estimated glomerular filtration rate

**Fig. 1:**
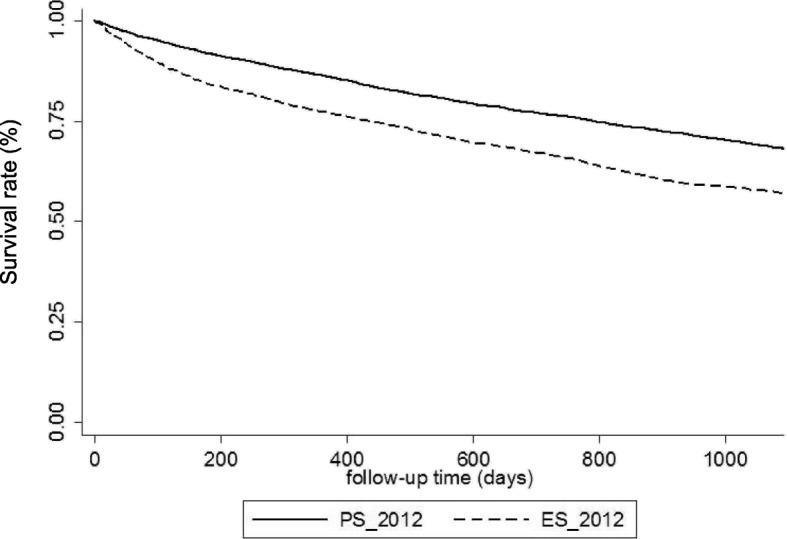
Kaplan Meier survival curves of dialysis incident patients in 2012 according to the dialysis initiation condition: planned start (PS_2012, full line) or emergency start (ES_2012, dashed line)

The three-year survival rate (Kaplan-Meier curves) was significantly lower in the ES than in the PS group (57% vs 68.2%, *p* &lt; 0.001) (Fig. 1). After adjustment for age, sex, comorbidities, nutritional status, pre-dialysis anemia management, baseline eGFR, vascular access and dialysis start condition (ES or PS), ES remained an independent risk factor of death within 3 years from dialysis start (Hazard Ratio 1.10, 95% CI 1.01–1.19) in the multivariate analysis (Table 2). Causes of death were similar in ES and PS groups (Additional file 1: Table S2). About first dialysis management, the use of central venous catheter was independently and strongly associated with worse three-year survival (HR 1.41, 95% CI 1.30–1.54).

**Table 2:** Factors associated with three-year mortality on multivariate analysis in 2012 incident patients (*n* = 8839)

Multivariate Cox model	HR	95% CI	p
Women (vs Men)	0.96	0.88–1.04	0.311
Age (vs 18–45 years)			
45–60	2.70	1.96–3.71	&lt; 0.001
60–75	4.99	3.70–6.73	&lt; 0.001
≥ 75	8.51	6.32–11.46	&lt; 0.001
Albumin &lt; 30 (vs ≥30 g/dl)	1.32	1.19–1.46	&lt; 0.001
Hemoglobin (vs [10–12] g/dl)			
&lt; 10	1.16	1.07–1.26	&lt; 0.001
&gt; 12	0.99	0.88–1.12	0.908
BMI (vs 23–25 kg/m^2^)			
&lt; 18.5	1.41	1.14–1.74	0.002
18.5–23	1.22	1.08–1.37	0.001
≥ 25	0.92	0.82–1.03	0.138
Diabetes (vs No)	1.09	1.01–1.18	0.025
Respiratory disease (vs No)	1.22	1.12–1.34	&lt; 0.001
Active malignancy (vs No)	1.76	1.60–1.93	&lt; 0.001
Hepatic disease (vs No)	2.01	1.66–2.43	&lt; 0.001
Cardiovascular diseases (vs 0)			
1	1.31	1.19–1.45	&lt; 0.001
2	1.54	1.39–1.72	&lt; 0.001
≥ 3	1.78	1.60–1.97	&lt; 0.001
Walking disability (vs Walk without help)			
Totally dependent for transfers	2.24	1.95–2.57	&lt; 0.001
Need assistance for mobility	1.70	1.55–1.86	&lt; 0.001
eGFR (vs [5–10]ml/min/1.73m^2^)			
&lt; 5	0.99	0.85–1.15	0.860
[10–15]	1.17	0.99–1.37	0.058
≥ 15	1.39	1.17–1.65	&lt; 0.001
1st RRT on PD (vs HD)	1.37	1.21–1.54	&lt; 0.001
1st RRT on catheter (vs No)	1.41	1.30–1.54	&lt; 0.001
Emergency start (vs planned start)	1.10	1.01–1.19	0.029

*BMI* Body Mass Index; Active malignancy: solid tumors or hematological malignancies; Cardiovascular diseases: myocardial infarction, arrhythmias, coronary insufficiency, heart failure, arteritis of the lower limbs, cerebrovascular accident; *RRT* Renal Replacement Therapy, *PD* Peritoneal Dialysis; *HD* Hemodialysis; *HR* Hazard Ratio; 95%*CI* 95%Confidence Interval

**Fig. 2:**
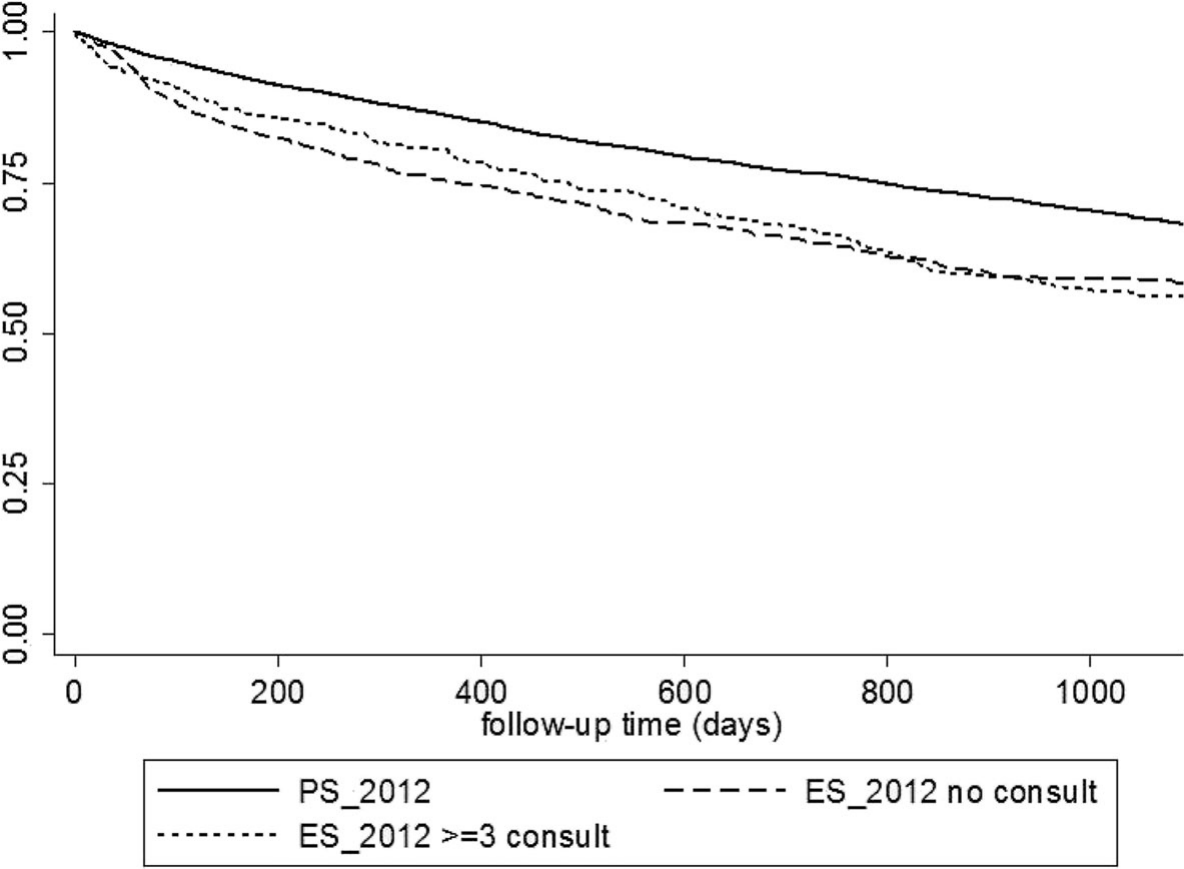
Kaplan Meier survival curves of dialysis incident patients in 2012 according to the previous care: ES with no previous consultation (ES_2012 no consult, dashed line), ES with more than two previous consultations (ES_2012 ≥ 3 consult, dotted line) or planned start (PS_2012, full line)

Some data provided insights into the patients’ management before dialysis start. In the ES group, 26.5% of patients were using an erythropoiesis-stimulating agent (ESA) and 20.5% had an arteriovenous fistula at dialysis initiation, thereby demonstrating a previous follow-up (Table 1). Moreover, among patients for whom the data was available (1240/2678), 36.4% of patients in the ES group visited the nephrologist three time or more in the year before dialysis initiation. The analysis of the characteristics of patients in the ES group with ≥3 previous visits (Table 3) revealed a particularly high proportion of patients with diabetes (53.4%), cardiovascular (54.3% had ≥2 cardiovascular diseases) and respiratory diseases (22.8%). Interestingly, 78.5% of them had a slowly progressive kidney disease, and they exhibited an ES with a median eGFR of 8.9 ml/min/1.73m^2^, close from patients of the PS group. The three-year survival rate was significantly lower in the ES group with ≥3 previous visits than in PS group (56.1% vs 68.2%, *p* &lt; 0.001) (Fig. 2). Conversely, patients in the ES group without previous nephrology consultation were younger and with less comorbidities, starting dialysis with low eGFR (median 6.2 ml/min/1.73m^2^); their three-year survival rate was 58.4%.

**Table 3:** Characteristics of patients in the ES group in 2012, according to the number of nephrology consultations in the year before starting dialysis

	No previous consultation 550/1240 (44.4%)	≥3 previous consultations 451/1240 (36.4%)	
	*n* (%)	*n* (%)	p
Men	360 (65.5)	316 (70.1)	0.121
Age, y (mean ± SD)	65 ± 18.06	69.2 ± 15.3	&lt; 0.001
Primary renal disease			&lt; 0.001
Acute nephropathy	145 (26.3)	31 (6.8)	
Slowly progressive nephropathy	236 (42.9)	354 (78.5)	
Unknown	169 (30.7)	66 (14.6)	
Serum Albumin &lt; 30 g/l	172 (31.3)	109 (24.2)	0.017
Missing	59 (10.7)	67 (14.9)	
BMI (kg/m^2^)			&lt; 0.001
&lt; 18.5	54 (9.8)	23 (5.1)	
18.5–25	219 (39.8)	154 (34.1)	
&gt; 25	186 (33.8)	213 (47.2)	
Missing	91 (16.5)	61 (13.5)	
Smoking status			0.003
Current smoker	91 (16.5)	45 (10)	
Former smoker	143 (26)	152 (33.7)	
Never smoker	216 (39.3)	165 (36.6)	
Missing	100 (18.2)	89 (19.7)	
Respiratory disease	72 (13.1)	103 (22.8)	&lt; 0.001
Hepatic disease	24 (4.4)	12 (2.7)	0.353
Active malignancy^a^	109 (19.8)	51 (11.3)	0.001
Diabetes	173 (31.5)	241 (53.4)	&lt; 0.001
Cardiovascular diseases^b^			&lt; 0.001
0	254 (46.2)	120 (26.6)	
1	125 (22.7)	86 (19.1)	
2	78 (14.2)	101 (22.4)	
≥ 3	93 (16.9)	144 (31.9)	
Mobility			0.262
Totally dependent for transfers	48 (8.7)	32 (7.1)	
Need assistance for transfers	84 (15.3)	57 (12.6)	
Walk without help	377 (68.5)	317 (70.3)	
First dialysis modality			0.027
HD/HDF	543 (98.7)	436 (96.7)	
APD/CAPD	7 (1.3)	15 (3.3)	
Vascular access at initiation			
Central venous catheter	537 (97.6)	301 (66.7)	&lt; 0.001
Arteriovenous fistula (used or not)	30 (5.5)	207 (45.9)	&lt; 0.001
Pre-dialysis anemia management			
Hemoglobin &lt; 10 g/dl	406 (73.8)	251 (55.7)	&lt; 0.001
ESA therapy	30 (5.5)	251 (55.7)	&lt; 0.001
Residual eGFR ml/min/1.73m^2^ (median, IQR)	6.2 (4.2–9.3)	8.9 (6.7–11.7)	&lt; 0.001
Missing	73 (13.3)	30 (6.7)	

^a^ Active malignancy: solid tumors or hematological malignancies

^b^Cardiovascular diseases: myocardial infarction, arrhythmias, coronary insufficiency, heart failure, arteritis of the lower limbs, cerebrovascular accident

*BMI* Body Mass Index*, HD* hemodialysis*, HDF* hemodiafiltration*, APD* automated peritoneal dialysis*, CAPD* continuous ambulatory peritoneal dialysis*, ESA* erythropoietin stimulating agent*, eGFR* estimated glomerular filtration rate

### Comparison between the 2012 and 2006 data

Next, data on 6119 patients who started dialysis in 2006 (92.6% of all incident patients in the 16 regions included in the REIN registry at that time) were compared with the data on the 7084 patients from the same regions who started dialysis in 2012.

ES proportion was similar in both years: 29.2% in 2012 and 28.4% in 2006 (*p* = 0.282). Nevertheless, the detailed comparison by region showed large local differences (Additional file 1: Table S3). Some regions did manage to reduce the ES percentage from 2006 to 2012 (e.g., in Lorraine, the ES rate dropped from 49.8% in 2006 to 19.5% in 2012).

The clinical profile of patients with ES remained similar, with high comorbidity level (Table 4). The proportion of some comorbidities were even higher in 2012 than in 2006: 17.6% of patients had a respiratory disease in 2012 vs. 14.1% in 2006 (*p* = 0.001), and 25.5% had serum albumin concentration &lt; 30 g/l in 2012 vs. 19.6% in 2006, *p* = 0.004.

**Table 4:** Characteristics of patients in ES group in 2006 and 2012 (16 French regions)

	ES 2006	ES 2012	
	*n* (%)	*n* (%)	p
Emergency start rate n (%)	1736/6119 (28.4)	2070/7084 (29.2)	0.282
Men	1060 (61.1)	1339 (64.7)	0.021
Age, y (mean ± SD)	67.2 ± 16.4	67.4 ± 16.6	0.62
Primary renal disease			0.55
Acute nephropathy	278 (16)	321 (15.5)	
Slowly progressive nephropathy	1032 (59.5)	1266 (61.2)	
Unknown	426 (24.5)	483 (23.3)	
Serum Albumin &lt; 30 g/l	340 (19.6)	528 (25.5)	0.004
Missing	535 (30.8)	487 (23.5)	
BMI (kg/m^2^)			0.026
&lt; 18.5	83 (4.8)	116 (5.6)	
18.5–25	584 (33.6)	603 (29.2)	
&gt; 25	581 (33.5)	749 (36.2)	
Missing	488 (28.1)	602 (29.1)	
Smoking status			0.002
Current smoker	168 (9.7)	205 (9.9)	
Former smoker	357 (20.6)	481 (23.2)	
Never smoker	966 (55.6)	981 (47.4)	
Missing	245 (14.1)	403 (19.5)	
Respiratory disease	244 (14.1)	364 (17.6)	0.001
Hepatic disease	41 (2.4)	65 (3.1)	0.146
Active malignancy^a^	217 (12.5)	303 (14.6)	0.056
Diabetes	666 (38.4)	853 (41.2)	0.145
Cardiovascular diseases^b^			0.763
0	705 (40.6)	840 (40.6)	
1	397 (22.9)	449 (21.7)	
2	290 (16.7)	348 (16.8)	
≥ 3	344 (19.8)	433 (20.9)	
Mobility			0.86
Totally dependent for transfers	166 (9.6)	190 (9.2)	
Need assistance for transfers	333 (19.2)	389 (18.8)	
Walk without help	1237 (71.3)	1491 (72)	
First dialysis modality			&lt; 0.001
HD/HDF	1683 (96.9)	2033 (98.2)	
APD/CAPD	53 (3.1)	37 (1.8)	
Vascular access at initiation			
Central venous catheter	1455 (83.8)	1782 (86.1)	0.001
Arteriovenous fistula (used or not)	419 (24.1)	432 (20.9)	0.016
Pre-dialysis anemia management			
Hemoglobin &lt; 10 g/dl	920 (53)	1218 (58.8)	0.001
ESA therapy	496 (28.6)	507 (24.5)	0.289
Residual eGFR ml/min/1.73m^2^ (median, IQR)	7.23 (5.1–10)	7.75 (5.4–10.7)	0.001
Missing	368 (21.2)	398 (19.2)	
Waitlisted at dialysis initiation (&lt; 80 years)	10 (0.7)	31 (2)	0.004

^a^ Active malignancy: solid tumors or hematological malignancies

^b^Cardiovascular diseases: myocardial infarction, arrhythmias, coronary insufficiency, heart failure, arteritis of the lower limbs, cerebrovascular accident

*BMI* Body Mass Index, *HD* hemodialysis*, HDF* hemodiafiltration*, APD* automated peritoneal dialysis*, CAPD* continuous ambulatory peritoneal dialysis*, ESA* erythropoietin stimulating agent*, eGFR* estimated glomerular filtration rate

## Discussion

This large epidemiologic study, based on prospectively collected data from the REIN registry shows that in France, the ES rate is very high (about 30% of all incident dialysis patients in 2012) and remained stable between 2006 and 2012. This result is consistent with studies on other European and North-American cohorts [15, 16] that highlighted the difficulty in reducing ES rate, despite the development of multidisciplinary management for ESRD [17].

Our study shows unequivocally that ES is associated with worse prognosis than PS (three-year survival: 57% for ES vs 68.2% for PS, *p* &lt; 0.001), indicating that ES deleterious effect is never overcome. Indeed, after full adjustment, ES was still an independent risk factor for death at 3 year **(**HR 1.10, 95% CI 1.01–1.19). Our results confirm previous studies in smaller cohorts of French dialyzed patients showing that ES is associated with early mortality on dialysis [16, 18]. Similarly, Descamp et al. demonstrated in a monocentric study that ES was the major confounding factor explaining the over-mortality of hemodialysis compared with peritoneal dialysis as first RRT modality, and ES was the only factor strongly associated with early mortality in dialysis [16]. Moreover, the STARRT study showed that the benefits of early referral to a nephrologist are lost in the case of “suboptimal” dialysis start (i.e., not starting with the planned modality or as an inpatient or with a central venous catheter) [12, 13]. Furthermore, ES is associated with poor quality of life and a substantial heavier healthcare burden [15].

Due to ES deleterious impact, it is important to precise the profile of ES patients, in order to improve their management. Our data bring some insights on the clinical profile of patients exhibiting an ES. Compared with patients in the PS group, these patients had higher comorbidity burden, especially cardiovascular diseases. Moreover, within the ES group, we could distinguish two discrete subgroups based on previous nephrology care: on one hand, patients without pre-dialysis care (no previous nephrology consultation), who experimented ES with low residual renal function because of an acute kidney injury or an undiagnosed ESRD. On the other hand, patients with consistent pre-dialysis care (≥3 consultations in the previous year), who probably initiated the RRT preparation (confirmed by arteriovenous fistula and the use of ESA), but presented a life-threatening event before the appearance of uremic symptoms. They mainly have slowly progressive nephropathy, but a particularly high comorbidity burden that could lead to an acute decompensation of their cardiovascular or respiratory condition at quite high eGFR. Patients in the ES group with ≥3 visits have worse three-year survival than patients in the PS group in our study (56.1% vs 68.2%, *p* &lt; 0.001).

These findings corroborate the results of the only other study on this topic, performed in 184 Canadian patients. In this study, the group of patients with previous follow-up but unplanned start had a significantly worse one-year survival rate than patients with planned first dialysis. Congestive heart failure and higher BMI were independently associated with the risk of unplanned dialysis start (i.e., initiated as inpatient) [17]. These and our findings suggest that the ESRD management of patients with high comorbidity burden needs to be improved and question the suitability of the current recommended strategy of dialysis initiation at the uremic symptomatic stage for these high-risk patients.

This is apparently in contradiction with the recent evidence-based guidelines on “late initiation”. However, these recommendations are based on the IDEAL cohort, a highly selected subgroup of younger, relatively “healthy” patients with a careful follow-up and, consequently, they may not fully apply to patients with high comorbidity burden. Controlled trials are needed to determine whether early dialysis initiation could avoid ES and improve survival in this specific group of patients. Another way of improvement is to optimize the attendance of pre-dialysis clinics, and to increase the ESRD follow-up frequency [19]. Indeed, Singhal et al. underlined that “cumulative care” (number of nephrology consultations) and “consistent critical period care” (defined as ≥3 consultations during the 6 months before dialysis initiation) are more relevant than the classic “early referral”, and are independently associated with better survival [20].

Even if a part of ES remains unavoidable (acute kidney injuries), reducing the proportion of ES is not a desperate cause: some regions in France managed to decrease ES rate from 2006 to 2012. For example, the Lorraine region set up a city-hospital nephrology network (Nephrolor) and increased the volume of information given to the general practitioners. ES proportion dropped from 49.8% in 2006 to 19.5% in 2012. This example indicates that ES high rate is not inevitable, but a proactive ESRD care policy is needed.

The main limitations of our study are linked to the nature of a registry-based epidemiologic work. First, the definition of “ES” remains open to criticism. The REIN registry classifies as ES any first dialysis occurring within 24 h after a nephrology consultation because of a life-threatening complication. The problem of not standardized terminology about unplanned dialysis start is still unsolved. Mendelssohn proposed the term of “suboptimal initiation”, defined as starting dialysis as an inpatient or with a central venous catheter, or not with the planned dialysis modality [15]. However, we consider this definition inappropriate because many patients, mostly elderly, are not eligible to arteriovenous fistula or refuse it, but may start dialysis on a catheter in a planned setting.

Another limitation concerns the “previous visit” item, which was added in the registry only in 2009. However, this information was available only for 45% (*n* = 2797) of all 2012 incident patients included in our study. Therefore, the conclusions based on this information must be interpreted cautiously. Finally, the REIN registry does not record the specific cause of dialysis start, which could be useful to better identify the patients’ profile and ES context.

## Conclusions

Our study shows that in mainland France, the absolute rate of ES is still high (about 30% of all incident patients) and didn’t decrease between 2006 and 2012. After full adjustment, ES remains independently associated with higher three-year mortality risk. A substantial proportion of ES was observed in patients with regular previous follow-up but high comorbidity burden, suggesting acute decompensation as the cause of ES. More data are needed to identify patients at risk of ES and improve their pre-dialysis management, in order to avoid ES and its deleterious consequences.

## Additional file

Additional file 1: **Table S1.** Factors associated with three-year mortality on univariate analysis in 2012 incident patients (imputed dataset). **Table S2.** Causes of death by dialysis initiation condition in 2012 patients. **Table S3.** Proportion of patients with emergency start per French region, in 2006 and 2012. **Appendix S1.** Classification of primary kidney nephropathies in the REIN registry depending on the form of renal function impairment. (PDF 109 kb)

## Abbreviations

BMI: Body Mass Index
CKD: Chronic Kidney Disease
eGFR: Estimated glomerular filtration rate
ES: Emergency start
ESA: Erythropoietin stimulating agent
ESRD: End Stage Renal Disease
PS: Planned start
REIN: Renal Epidemiology and Information Network
RRT: Renal replacement therapy
