# Correction to: Deleterious effects of dialysis emergency start, insights from the French REIN registry

**DOI:** 10.1186/s12882-018-1063-6

**Published:** 2018-10-15

**Authors:** Alain Michel, Adelaide Pladys, Sahar Bayat, Cécile Couchoud, Thierry Hannedouche, Cécile Vigneau

**Affiliations:** 1 CHU Pontchaillou, Service de néphrologie, 2 rue H Le Guilloux, 35033 Rennes, France; 2 EHESP, Département d’Epidémiologie et de Biostatistiques, Rennes, France; 3 Université Rennes 1, UMR CNRS, 6290 Rennes, France; 4 EA MOS EHESP, Rennes, France; 5 Registre REIN, Agence de la biomédecine, La Plaine Saint Denis, France; 6 Faculté de médecine de Strasbourg, Hôpitaux universitaires de Strasbourg, 1 place de l’Hôpital, 67091 Strasbourg, France; 7 Université de Rennes 1, 2 av prof L Bernard, 35000 Rennes, France; 8 Inserm (Institut national de la santé et de la recherche médicale), IRSET, U1085, SFR Biosit, 9 Avenue du Professeur Léon Bernard, 35000 Rennes, France

## Correction

Following publication of the original article [1], the authors reported that all of the authors’ names were processed incorrectly so that their given and family names were interchanged. In this Correction the correct author names are shown. The original publication of this article has been corrected.

The correct names are: Alain Michel, Adelaide Pladys, Sahar Bayat, Cécile Couchoud, Thierry Hannedouche and Cécile Vigneau.
