# Supplementary material for: Deleterious effects of dialysis emergency start, insights from the French REIN registry
Source: BMC Nephrol. 2018 Sep 17;19:233. doi: 10.1186/s12882-018-1036-9 (PMC6142323; doi:10.1186/s12882-018-1036-9)
Supplement: Supplementary file 1 — Table S1. Factors associated with three-year mortality on univariate analysis in 2012 incident patients (imputed dataset). Table S2. Causes of death by dialysis initiation condition in 2012 patients. Table S3. Proportion of patients with emergency start per French region, in 2006 and 2012. Appendix S1. Classification of primary kidney nephropathies in the REIN registry depending on the form of renal function impairment. (PDF 109 kb) [file 12882_2018_1036_MOESM1_ESM.pdf]

Table S1. Factors associated with three-year mortality on univariate analysis in 2012 incident patients (imputed dataset).

**Univariate Cox model**	**HR**	**95% CI**	**p**
**Women** (vs Men)	0.91	0.84-0.98	0.011
**Age** (vs 18-45 years)			
45-60	2.97	2.17-4.07	<0.001
60-75	6.90	5.15-9.25	<0.001
≥75	13.02	9.75-17.4	<0.001
**Albumin** <30 (vs ≥30 g/dl)	1.81	1.66-1.96	<0.001
**Hemoglobin** (vs [10-12] g/dl)			
<10	1.23	1.14-1.33	<0.001
>12	0.97	0.86-1.10	0.657
**Smoking status** (vs never smoker)			
Current/former smoker	1.13	1.05-1.23	0.002
**BMI** (vs 23-25 kg/m²)			
<18.5	1.17	0.97-1.40	0.092
18.5-23	1.14	1.02-1.29	0.025
≥25	0.92	0.83-1.02	0.126
**Diabetes** (vs No)	1.29	1.20-1.39	<0.001
**Respiratory disease** (vs No)	1.82	1.66-1.98	<0.001
**Active malignancy** (vs No)	2.12	1.93-2.32	<0.001
**Hepatic disease** (vs No)	1.98	1.65-2.38	<0.001
**Cardiovascular diseases** (vs 0)			
1	1.91	1.73-2.11	<0.001
2	2.68	2.42-2.97	<0.001
≥ 3	3.37	3.06-3.71	<0.001
**Walking disability** (vs Walk without help)			
Totally dependent for transfers	3.79	3.33-4.31	<0.001
Need assistance for mobility	2.80	2.56-3.07	<0.001
**eGFR** (vs [5-10[ml/min/1.73m²)			
<5	1.25	1.08-1.45	0.003
[10-15[	1.68	1.44-1.95	<0.001
≥15	2.42	2.07-2.84	<0.001
**1st RRT on PD** (vs HD)	1.08	0.96-1.21	0.213
**1st RRT on catheter** (vs No)	1.91	1.77-2.06	<0.001
**Emergency start** (vs planned start)	1.50	1.40-1.61	<0.001

BMI: Body Mass Index; Active malignancy: solid tumors or hematological malignancies; Cardiovascular diseases: myocardial infarction, arrhythmias, coronary insufficiency, heart failure, arteritis of the lower limbs, cerebrovascular accident; RRT: Renal Replacement Therapy; PD: Peritoneal Dialysis; HD: Hemodialysis; HR: Hazard Ratio; 95%CI: 95%Confidence Interval.

Table S2. Causes of death by dialysis initiation condition in 2012 patients

Causes of death	2012 PS n (%)	2012 ES n (%)
**Cardiovascular diseases**	450 (22.9)	248 (21.6)
**Renal diseases**	19 (1.0)	11 (1.0)
**Active malignancy**	218 (11.1)	134 (11.7)
**Diabetes**	3 (0.2)	1 (0.1)
**Infectious disease**	232 (11.8)	155 (13.5)
**Cachexia**	135 (6.9)	77 (6.7)
**Hyperkaliema**	20 (1.0)	9 (0.8)
**Hepatic disease**	17 (0.9)	3 (0.3)
**Unexpected death**	187 (9.5)	102 (8.9)
**Unknown**	424 (21.6)	251 (21.8)
**Other causes**	257 (13.1)	159 (13.9)
Total	1962 (100)	1150 (100)

Table S3. Proportion of patients with emergency start per French region, in 2006 and 2012.

**French region**	**2006** N (%)	**2012** N (%)
Alsace	-	96 (31.3)
Aquitaine	-	236 (50.4)
Auvergne	59 (34.1)	45 (21.3)
Basse-Normandie	21 (12.4)	35 (20.3)
Bourgogne	44 (21.9)	40 (16.7)
Bretagne	83 (25.2)	80 (19.8)
Centre	102 (27.4)	175 (50.7)
Champagne-Ardenne	80 (34.5)	51 (24.2)
Corse	19 (46.3)	4 (15.4)
Franche-Comté	-	25 (18.8)
Haute-Normandie	60 (31.3)	59 (25.9)
Ile-de-France	268 (19.8)	451 (26.1)
Languedoc-Roussillon	119 (26.7)	151 (31.8)
Limousin	16 (16.2)	29 (26.1)
Lorraine	147 (49.8)	69 (19.5)
Midi-Pyrénées	80 (21.9)	117 (30.2)
Nord-Pas-de-Calais	207 (38.2)	263 (36.6)
Pays de la Loire	-	101 (25.1)
Picardie	-	101 (40.9)
Poitou-Charentes	-	49 (24.7)
Provence-Alpes-Côte-d'Azur	205 (28.1)	244 (33.4)
Rhône-Alpes	226 (38.7)	257 (34.6)
Total	1736 (28.4)	2678 (30.3)

Appendix: *Classification of primary kidney nephropathies in the REIN registry depending on the form of renal function impairment:*

**Acute nephropathy**<br>**(including CKD exacerbation/flare)**	**Slowly progressive nephropathy**	**Unknown**
Amyloidosis<br>Extracapillary glomerulonephritis<br>Cryoglobulinemia<br>Wegener's granulomatosis<br>Acute kidney Injury<br>Other systemic disease<br>Myeloma<br>Ischemic nephropathy, cholesterol Embolic disease<br>Lupus nephropathy<br>Vascular nephropathy due to malignant hypertension<br>Traumatic loss of kidney<br>Rheumatoid purpura<br>Systemic scleroderma<br>Goodpasture syndrome<br>Thrombotic microangiopathy	Kidney agenesis, hypoplasia, dysplasia<br>Morphological abnormality<br>Diabetes<br>Glomerulonephritis with FSGS<br>Membranous glomerulonephritis<br>Membranoproliferative glomerulonephritis<br>Kidney infection<br>Hereditary kidney disease<br>Tubulointerstitial nephritis due to neurological bladder<br>Tubulointerstitial nephritis secondary to lithiasis<br>Tubulointerstitial nephritis due to acquired obstructive uropathy<br>Tubulointerstitial nephritis due to congenital obstructive uropathy<br>Other tubulointersitial nephritis<br>Nephrocalcinosis<br>CKD due to vesicoureteral reflux<br>Hereditary nephropathy with deafness<br>Vascular nephropathy due to hypertension<br>IgA nephropathy<br>Primary oxalosis<br>Cystic disease<br>Polycystic kidney<br>Renal tuberculosis<br>Tubulopathies (Dent, Lowe, Barter…)<br>Renal or urinary tract cancer	Glomerulonephritis without histological examination<br>Glomerulonephritis with another histological diagnosis<br>Secondary glomerular nephritis<br>Unknown<br>Vascular nephropathy from other cause<br>Vascular nephropathy, unprecise cause<br>Toxic nephropathy

CKD: chronic kidney disease; FSGS: focal segmental glomerulosclerosis, IgA: immunoglobulin A.
